# The impact of the COVID-19 pandemic on patients awaiting spinal cord stimulation surgery in the United Kingdom: a multi-centre patient survey

**DOI:** 10.1177/2049463720948092

**Published:** 2020-08-21

**Authors:** Ganesan Baranidharan, Beatrice Bretherton, Sam Eldabe, Vivek Mehta, Simon Thomson, Manohar Lal Sharma, Girish Vajramani, Stana Bojanic, Ashish Gulve, James FitzGerald, Samuel Hall, Julie Firth

**Affiliations:** 1 Leeds Pain and Neuromodulation Centre, Leeds Teaching Hospitals NHS Trust, Leeds, UK; 2 School of Medicine, Faculty of Medicine and Health, University of Leeds, Leeds, UK; 3 School of Biomedical Sciences, Faculty of Biological Sciences, University of Leeds, Leeds, UK; 4 Department of Pain Medicine, The James Cook University Hospital, Middlesbrough, UK; 5 Pain and Anaesthesia Research Centre, St Bartholomew’s Hospital, London, UK; 6 Department of Anaesthesiology, Basildon and Thurrock University Hospitals, Basildon, UK; 7 Department of Pain Medicine, The Walton Centre NHS Foundation Trust, Liverpool, UK; 8 Wessex Neurological Centre, University Hospital Southampton, Southampton, UK; 9 Department of Neurosurgery, The John Radcliffe Hospital, Oxford, UK; 10 Nuffield Department of Surgical Sciences, University of Oxford, Oxford, UK

**Keywords:** COVID-19, spinal cord stimulation, chronic pain, mental health, telephone survey

## Abstract

**Introduction:**

Spinal cord stimulation (SCS) is a recommended treatment for chronic refractory neuropathic pain. During the COVID-19 pandemic, elective procedures have been postponed indefinitely both to provide capacity to deal with the emergency caseload and to avoid exposure of elective patients to COVID-19. This survey aimed to explore the effect of the pandemic on chronic pain in this group and the views of patients towards undergoing SCS treatment when routine services should resume.

**Methods:**

This was a prospective, multi-centre telephone patient survey that analysed data from 330 patients with chronic pain who were on an SCS waiting list. Questions focussed on severity of pain, effect on mental health, medication consumption and reliance on support networks during the COVID-19 pandemic. Views towards undergoing SCS therapy were also ascertained. Counts and percentages were generated, and chi-square tests of independence explored the impact of COVID-19 risk (very high, high, low) on survey responses.

**Results:**

Pain, mental health and patient’s ability to self-manage pain deteriorated in around 47%, 50% and 38% of patients, respectively. Some patients reported increases in pain medication consumption (37%) and reliance on support network (41%). Patients showed a willingness to attend for COVID-19 testing (92%), self-isolate prior to SCS (94%) and undergo the procedure as soon as possible (76%).

**Conclusion:**

Our findings suggest that even during the COVID-19 pandemic, there remains a strong clinical need for patients with chronic pain identified as likely SCS responders to be treated quickly. The current prioritisation of new SCS at category 4 (delayed more than 3 months) is challenged judging by this national survey. These patients are awaiting SCS surgery to relieve severe intractable neuropathic pain. A priority at category 3 (delayed up to 3 months) or in some selected cases, at category 2 are the appropriate priority categories.

## Introduction

In late December 2019, a new strain of coronavirus, later called COVID-19, was identified in Wuhan, China. By 30 January 2020, COVID-19 was classed as a public health emergency of international concern (PHEIC) by the World Health Organization and declared a pandemic on 11 March 2020. Currently recognised symptoms of COVID-19 include fever, cough, anosmia and shortness of breath and, in severe cases, breathing difficulties which can require mechanical ventilation. Complications of COVID-19 have been reported to include pneumonia, adult respiratory distress syndrome, cytokine storm and sepsis.

As of the 17 June 2020, the United Kingdom had 299,600 confirmed cases and 42,054 deaths, although it is thought the actual rates of cases and deaths may be higher.^1^ In the United Kingdom, COVID-19 has had profound effects on National Health Service (NHS) services, particularly following the country’s lockdown from the 23 March 2020. This resulted in the UK department of health instructing NHS Trusts to discontinue most elective operating from the 15 April 2020 for a period of at least 3 months.^2^ The UK intercollegiate guidance issued re-prioritisation of surgical procedures and the careful planning of how to recover surgical services following the easing of COVID-19 preventive measures.^3,4^ Due to the unprecedented nature of COVID-19, it is unclear how the disease and preventive measures have impacted upon patient’s symptoms and care.

Chronic pain is characterised by severe pain that continues for longer than 12 weeks despite medication or treatment. It has negative effects on quality of life and is a contributing factor to mental health problems. The prevalence of mood and anxiety disorders has been reported to range from 1% to 61% in chronic pain conditions.^5^ Prevalent opioid prescribing for non-cancer pain^6^ can lead to hypogonadism^7^ and substance abuse disorders; indeed 1–43% of patients with chronic pain have an opioid use disorder.^5^ Social isolation and loneliness are also thought to result from chronic pain, as well as be contributing factors to its emergence.^8–10^ Therefore, given the anxiety surrounding COVID-19,^11^ immunosuppressant effects of opioid administration^12,13^ and association between social isolation and chronic pain,^8–10^ the COVID-19 pandemic may present significant challenges to individuals with chronic pain conditions.

Spinal cord stimulation (SCS) is an National Institute for Health and Care Excellence (NICE)-recommended procedure for intractable chronic pain of neuropathic origin (TA159) and is provided in around 28 centres in the United Kingdom as a specialist service.^14^ SCS significantly reduces pain in individuals with failed back surgery syndrome (FBSS),^15^ complex regional pain syndrome (CRPS)^16,17^ and painful diabetic neuropathy.^18^ Health-related quality of life and pain-related disability improve with SCS.^19^ Also, decreases in medication consumption, such as opioids and anti-neuropathic medication, have been evidenced in research exploring the efficacy and safety of SCS.^20,21^ SCS can benefit patients who have failed other treatments.

Although not explicitly stated, National Health Service England (NHSE) guidelines for the prioritisation of surgical procedures during the COVID-19 pandemic suggest that SCS be considered priority category 4, although in certain clinical scenarios it could be appropriately categorised as category 2 or 3. This ambiguity in priority level may influence provider decision-making, and result in unacceptable and unfair delays to chronic pain patients awaiting SCS; which could have a profound impact on the physical and mental needs of a patient suffering with anxiety, severe pain and a tendency to drug misuse.

Undergoing surgery during the COVID-19 pandemic is not without serious risks. Indeed, using mathematic modelling, of 91,410 elective surgery outpatients, at least 75.90 (near 1%) would develop preventable patient infections in Washington State alone if elective outpatient procedures continued as normal.^22^ In addition, in a sample of 34 patients who underwent elective surgeries during the incubation period of COVID-19, all developed COVID-19 pneumonia shortly after surgery.^23^ The complexity of surgery also appears to be an important factor: the more complex the surgery, the greater the number of COVID-19 patients admitted to the intensive care unit (ICU).^23^ Furthermore, 30-day mortality has been estimated at around 24% for elective and emergency surgery,^24^ with the risk of mortality from COVID-19 pneumonia in the perioperative period being near 21% for procedures done both under general and local anaesthesia.^23^ However, it is important to note that these data are not specific to a particular group of patients or type of surgery, rendering it unclear how much variability there is between clinical conditions and procedures for COVID-19 risk. In response, our study characterised the willingness of chronic neuropathic pain patients to undergo a neuromodulation procedure (SCS) and how chronic pain patients view the balance of risk of COVID-19 infection versus the potential benefit of SCS.

## Methods

This was a prospective, multi-centre telephone patient survey conducted during May 2020 in seven UK centres. The study received local audit board registration. All centres used the same questionnaire (see Supplementary Section). This enabled us to obtain a national picture of patients who are currently waiting for SCS therapy.

### Aims

We aimed to survey patients on the waiting list to undergo an SCS implant to seek their views on the impact of the pandemic and lockdown on their chronic pain and their willingness to attend an NHS hospital for SCS surgery.

### Participants and methods

We set out to contact all patients waitlisted for a neuromodulation procedure (SCS) for chronic pain, at seven NHS hospitals. A small percentage of patients were waiting to undergo surgical paddle lead implantation. No formal sample size calculation was conducted. Four hundred and three adults on an SCS waiting list were contacted by telephone to take part in the survey of which 330 responded.

Briefly, the survey elicited responses from participants on questions related to their personal risk of mortality from COVID-19, the impact of lockdown on their pain, mental health and intake of analgesia and their willingness to attend their local hospital for an SCS implant. COVID-19 risk was ascertained for each participant. This was achieved using NHSE risk criteria (see Supplementary Section) and categorising participants as either very high, high or low COVID-19 risk. Participants were also asked if they had experienced COVID-19 symptoms or been formally tested for the disease and outcome of the test.

All survey responses were fully anonymised at individual centres and results collated centrally at the Leeds Pain Research Centre in a single MS Excel spreadsheet (G.B.). Data were analysed in SPSS (version 25) by a single researcher (B.B.) and counterchecked (G.B.). To analyse the data, counts and percentages were generated. To explore the impact of COVID-19 risk (very high risk, high risk, low risk) on survey responses, chi-square tests of independence were undertaken. An alpha level of 0.05 was deemed statistically significant.

### The SCS pathway

NICE TA159 recommends SCS for adults who have chronic neuropathic pain (measuring at least 50 mm on a 0–100 mm visual analogue scale) for at least 6 months despite more conservative management with medication and targeted injection treatment. SCS pathways vary between patients in order to address their individual needs. Briefly, the pathway involves assessment for suitability followed by information session, psychology assessment and multidisciplinary team (MDT) discussion. Opioid reduction may also be included, depending on the patient and unit. Patients are then placed on a waiting list for surgery. Surgery involves a temporary trial or a permanent trial.^25^ For the temporary trial, the trial wires are removed and patients relisted for a full implant. The permanent trial involves connecting the battery to the wires at the end of the trial period.

## Results

Four hundred and three patients on an SCS waiting list were contacted and 330 (n = 190 females) took part in the survey (82% response rate). The demographic characteristics of the participants are displayed in Table 1. The survey population included 56 patients who were very high COVID-19 risk, 118 who were high risk and 129 who were low COVID-19 risk. COVID-19 risk data were missing for 27 patients. Seven patients had received a COVID-19 test, with six confirming positive results. Twenty-nine patients had experienced signs and symptoms of COVID-19, but not tested.

**Table 1.** Summary of initial characteristics.

Demographic details	Total sample (n)	330
	Females (n)	190
	Age (mean ± SD, years)	53.56 ± 12.96
COVID-19 risk	Very high (n)	56
	High (n)	118
	Low (n)	129
	Missing data (n)	27
COVID-19 testing	Not tested (n)	186
	Tested positive (n)	6
	Tested negative (n)	1
	Missing data (n)	137
COVID-19 signs/symptoms	No signs/symptoms (n)	164
	Had signs/symptoms (n)	29
	Missing data (n)	137

### Pain and mental health deteriorated for most patients

**Figure 1.**
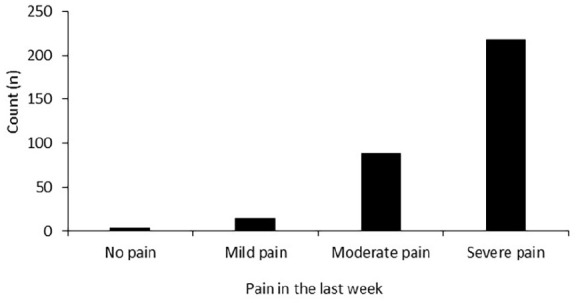
Patient reported pain severity in the 7 days prior to telephone interview.

The majority (n = 218, 67%) experienced severe pain in the previous week (see Figure 1). 27% (n = 88) reported moderate pain, with small numbers stating their pain was mild or not present (n = 15, 5%; n = 3, 1%; respectively). There were missing data for six patients.

**Figure 2.**
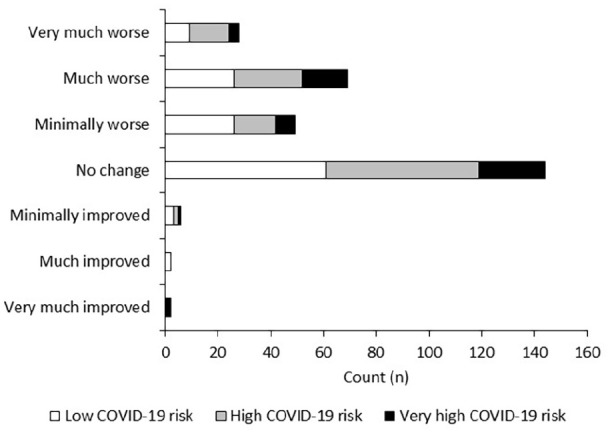
Change in patient reported pain severity during the COVID-19 pandemic in patients with chronic pain who were on an SCS waiting list. Change in pain was not significantly associated with COVID-19 risk (p > 0.05).

When asked how their pain had changed during the COVID-19 pandemic, few patients reported improvements in their pain (n = 10, 3%), with 50% (n = 162) of patients reporting no change in pain. Deteriorations in pain were reported in 155 patients (47%), with 72 (22%) stating pain was much worse compared to before COVID-19. This was followed by 53 (16%) and 30 patients (9%) who reported pain was minimally worse and very much worse, respectively. There were missing data for three patients. Change in pain was independent of COVID-19 risk (p > 0.05, see Figure 2).

**Figure 3.**
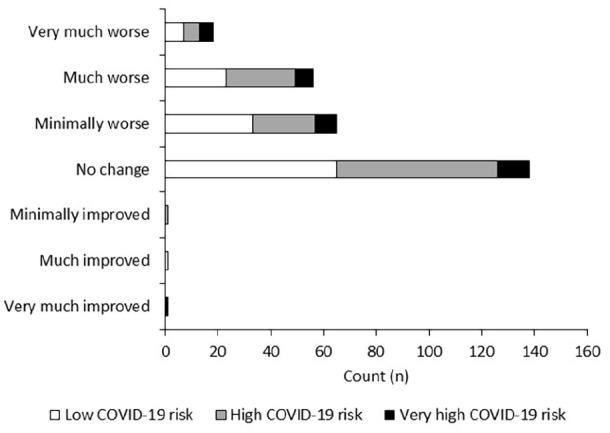
Change in patient reported mental health during the COVID-19 pandemic in patients with chronic pain who were on an SCS waiting list. This was not influenced by COVID-19 risk (p > 0.05).

With respect to mental health, only three patients reported improvements of any magnitude (minimally improved: n = 1; much improved: n = 1; very much improved: n = 1). However, 139 patients (50%) reported deteriorations in mental health (see Figure 3). This comprised 65 patients (23%) who had minimal deteriorations, 56 (20%) who reported their mental health was much worse and 18 patients (6%) who stated their mental health was very much worse. A similar number of patients reported no change in their mental health (n = 138, 49%). There were missing data on 50 patients as this was not collected in one centre due to local restrictions. Change in mental health was not significantly associated with COVID-19 risk (p > 0.05).

Change in pain was significantly related to change in mental health (p < 0.001, see Table 2). This was particularly driven by patients who reported ‘pain was similar to prior to the pandemic’, as these patients also reported ‘mental health had stayed the same’.

**Table 2.** Relationship between change in patient reported mental health and change in patient reported pain (p < 0.001). The shaded boxes reflect consistent responses between both categories. Data are presented raw.

		Mental health						
		Very much worse	Much worse	Minimally worse	Same	Minimally improved	Much improved	Very much improved
Pain	Very much worse	8	10	5	5	0	0	0
	Much worse	6	22	13	21	0	0	0
	Minimally worse	2	14	13	17	0	0	0
	Same	2	10	32	87	1	0	0
	Minimally improved	0	0	2	3	0	0	0
	Much improved	0	0	0	1	0	1	0
	Very much improved	0	0	0	1	0	0	1

### Some patients effectively self-managed their pain

**Figure 4.**
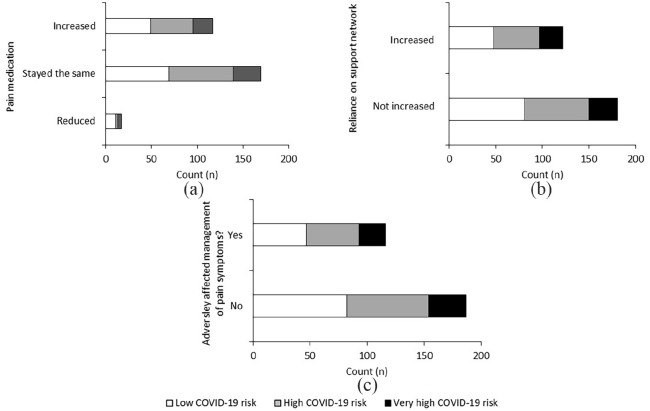
Change in pain medication consumption (a), change in reliance on support network (b), and management of pain symptoms (c) in patients with chronic pain who were on an SCS waiting list. These were not influenced by COVID-19 risk (p > 0.05).

For most patients, pain medication consumption stayed the same (n = 190, 58%, see Figure 4(a)). However, there was a sub-group who either reported increases in pain medication consumption (n = 121, 37%) or decreases (n = 19, 6%). Although 135 patients (41%) reported increased reliance on support networks during the lockdown due to increased pain severity (see Figure 4(b)), the majority of patients (n = 195) reported that reliance on a support network had not increased. In total, 124 patients reported that the COVID-19 pandemic had adversely affected their ability to manage pain symptoms (38%, see Figure 4(c)). Surprisingly, 206 patients (62%) stated the pandemic had not adversely affected the management of their pain symptoms. COVID-19 risk was not significantly associated with changes in pain medication, reliance on support network or the self-management of pain symptoms (p > 0.05).

### Strong clinical need for SCS during the COVID-19 pandemic

**Figure 5.**
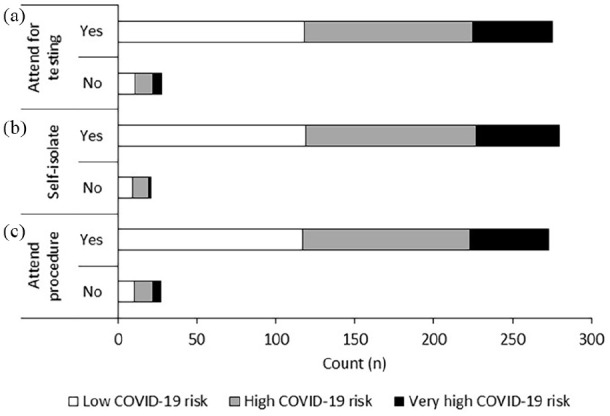
Number of patients who (a) would/would not attend for COVID-19 testing, (b) self-isolate after testing and prior to the procedure, and (c) attend hospital for the procedure. Responses were independent of COVID-19 risk (p > 0.05).

Reponses to question 13 in the survey (see Supplementary Section for the survey) demonstrated a high willingness from patients to undergo SCS surgery and adhere to COVID-19 related processes. 302 patients (92%) were willing to attend for COVID-19 swabs (see Figure 5), 307 (of 328, 94%) said they would be happy to self-isolate after COVID-19 testing and prior to the procedure (see Figure 5) and the majority of patients (n = 300, of 327, 92%) reported a willingness to attend for surgery (see Figure 5c).

**Figure 6.**
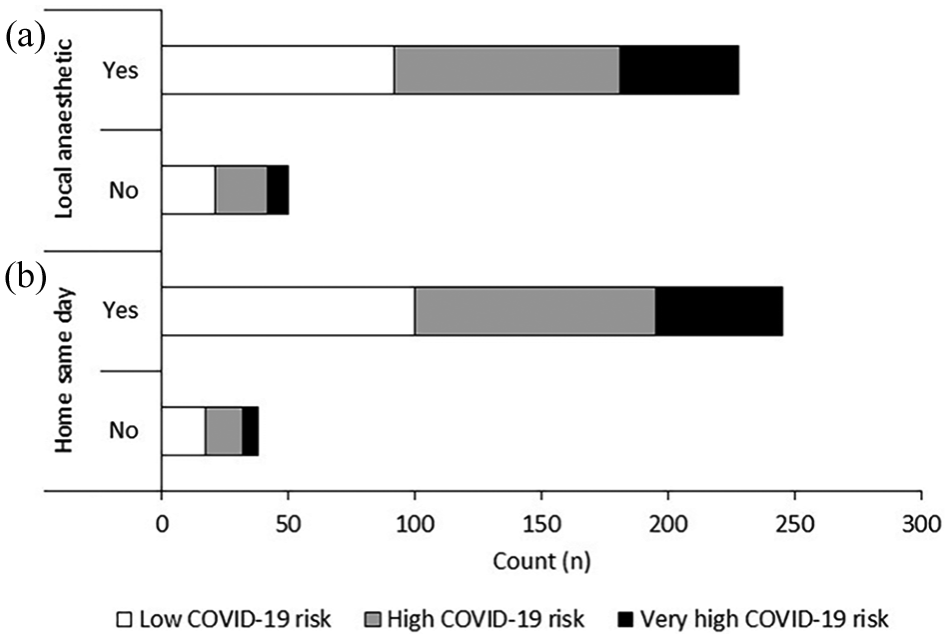
Number of patients who would/would not be willing to have a local anaesthetic and be sent home on the same day as the procedure. Responses were independent of COVID-19 risk (p > 0.05).

Most patients (251 of 305, 82%) said they would be willing to undergo the procedure with local anaesthetic with the option of being administered strong painkillers (see Figure 6a). When informed the procedure would take part in the non-COVID part of the hospital, 268 of 310 patients (86%) said they would prefer to go home on the same day of the procedure (see Figure 6b). These responses were independent of COVID-19 risk (p > 0.05).

**Figure 7.**
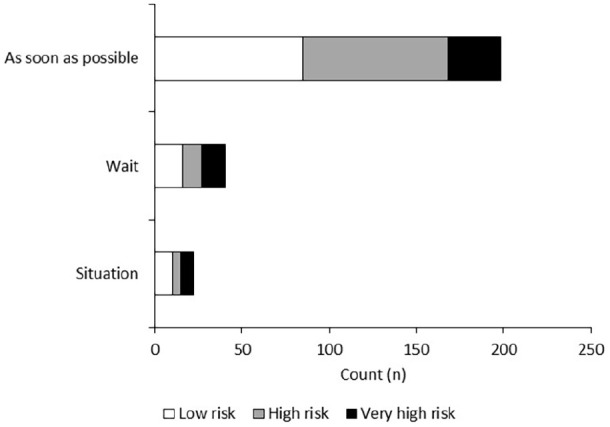
Number of patients who would prefer to have their procedure as soon as possible, wait until restrictions are lifted and evaluate the situation when a date is set for surgery.

When asked about the length of time patients would prefer to wait prior to having their SCS procedure, most patients (n = 220, 76%) stated a preference for having the procedure as soon as possible (see Figure 7). Forty-five patients (16%) said they would prefer to wait, particularly until the lockdown was lifted, with a small percentage of patients (n = 22, 8%) stating they would evaluate the situation when provided with a date of surgery. Two patients stated they no longer wanted SCS and there were missing data from 41 patients. Responses were independent of COVID-19 risk (p > 0.05).

## Discussion

To our knowledge this is the first prospective, multi-centre telephone survey that examined how the COVID-19 pandemic has impacted chronic pain patients awaiting SCS therapy in the United Kingdom. Findings revealed that the COVID-19 pandemic was associated with deteriorations in pain (47% of patients), mental health (50%) and ability to self-manage pain (38%). In addition, there was a sub-group of patients who reported increases in pain medication consumption (37%) and reliance on support network (41%). However, with the prospect of surgery, most patients said they would be willing to attend for COVID-19 testing (92%) and self-isolate prior to the procedure (94%), with 76% of patients stating a preference to undergo surgery as soon as possible. These findings therefore suggest that despite the risks posed by COVID-19, there is a strong clinical demand for SCS in patients with chronic pain, likely driven by severity of pain and challenges with mental health.

### Pain and mental health were negatively impacted by COVID-19

Deteriorations in pain and mental health occurred in some patients as a result of the COVID-19 pandemic. The direct healthcare costs associated with neuropathic pain in the United Kingdom is estimated to be around €2951 per patient per year.^26^ This includes costs attributed to consultations, drugs, surgical procedures, non-surgical procedures and alternative therapies.^26^ Within 2 weeks of the outbreak of COVID-19 in China, it was reported that around 54% of respondents to an online survey rated the psychological impact of the outbreak as moderate or severe.^11^ In addition, moderate to severe depressive, anxiety and stress symptoms were reported by 17%, 29% and 8% of respondents, respectively.^11^ Chronic pain patients are at a high risk for developing depression,^27^ social isolation can be a precursor to depression^28,29^ and countries were shut down to stem the spread of COVID-19. Therefore, the effects of the COVID-19 pandemic could have devastating consequences for the mental health of individuals with chronic pain if current lack of access to SCS persists. Indeed, findings revealed an association between change in pain and change in mental health, suggesting the two are closely linked. With the continuation of the NHS Mental Health Implementation Plan, it is hoped that these patients will be able to receive the psychological support they need in order to effectively self-manage their pain and prevent further impairment of their clinical condition and quality of life.

Although some patients were relatively effective at self-managing their pain (i.e. pain medication consumption stayed the same and reliance on support networks did not increase), this was not the case in other patients. Indeed, around 37% of patients reported increases in pain medication consumption. These figures are concerning given that opioids may theoretically mask or delay the detection of symptoms of COVID-19, such as myalgia, cough and shortness of breath.^30^ In addition, the immunosuppressant effects of chronic opioid treatment may potentially increase COVID-19 risk.^13,31^ As prolonged analgesia use has been associated with elevated mortality risk in osteoarthritis patients,^32^ the cardiac and COVID-19 risks of SCS patients need to be carefully considered and quantified. This is compounded by limited clarity concerning the association between high doses of opioids, immune suppression and COVID-19. Therefore, the prioritisation of patients for SCS implant should take into account changes in pain medication that have occurred since the beginning of the COVID-19 pandemic.

Around 41% of patients reported increased reliance on support networks during the lockdown due to increased pain severity. Enhanced carer burden/informal care has been associated with increases in societal costs. Indeed, annual professional caregiver costs are reported to be in the region of €1242 in the United Kingdom, but this is thought to constitute a small proportion of total care as most care is provided informally by friends and/or family.^26^ One study, for example, assessing broader cost of illness in chronic pain patients estimated mean annual societal costs to be €10,191 per year.^33^ As many workers have been furloughed or made redundant during the pandemic, the working lives of these patients may have been disrupted, in turn having further negative effects on the economic costs associated with their care. Productivity loss is also an element of the societal costs documented above; accounting for approximately 40% of costs.^33^ Worsening of symptoms may further impact a patient’s ability to work, compounding these productivity losses, and may also have a significant impact on the patient in a time of employment uncertainty. SCS is an effective treatment that may support an individual’s ability to work, and this may be an additional consideration concerning timely access. Further research investigating methods that influence these costs (e.g. timely intervention) may be warranted.

### A clinical need for SCS during the COVID-19 pandemic

The COVID-19 pandemic presents numerous risks to patients which may provoke depression, anxiety and stress.^11^ Despite these risks, most patients, including very high COVID-19 risk patients, stated a strong willingness to attend for SCS therapy, showing a preference for the surgery to happen as soon as possible. This therefore demonstrates a high clinical need for SCS in chronic pain waiting list patients, perhaps due to the humanistic impacts of the condition: enjoyment of life, mood, activity, work, relationships, sleep and employment are significantly impacted.^34^ In addition, pain profoundly disrupts everyday functioning (Brief Pain Inventory (BPI) score: 4.80) and has been associated with low quality of life (EuroQol-5D (EQ-5D) score: 0.57).^26,35,36^ Quality-adjusted life years (QALY) loss has been reported to be 64% in patients with chronic discogenic low back pain with around 98% (78/80) of patients showing some physical limitations.^37^ To more fully understand how COVID-19 impacts the presentation of chronic pain, validated questionnaires that quantify pain, including the contribution of neuropathic elements, pain-related disability, quality of life, QALY and the impact of pain on daily functioning, should be employed. These data could then be used to stratify patients and prioritise those who are in urgent need of therapy and can safely undergo the procedure. Although there is little data pertaining to the willingness of patients to attend for non-mortality related interventions, with increasing research into the impact of COVID-19 in other specialities, it is hoped that comparisons will be possible in time.

With the gradual easing of rules surrounding COVID-19, the reuptake of elective surgeries needs to be carefully managed and monitored. Given delays in treatment have occurred for some patients during the COVID-19 pandemic, these impacts need to be comprehensively assessed. Indeed, in normal times, delays to treatment add avoidable costs to the patient journey. For instance, for every 1-year increase in the time between a chronic pain diagnosis to SCS implantation, the likelihood of consuming more opioids, having more hospitalisations, healthcare appointments and medical expenditures increases.^38^ Furthermore, the quality of communication between healthcare professionals and patients, combined with reports in the media, will profoundly modulate how patients perceive their current and prospective treatment. This therefore calls for future research to systematically monitor the effects of delayed treatment over the long term. This could include evaluating the effectiveness of the e-tool:^39^ a new clinical resource to support referrers and implanters in optimising patient flow into secondary care through supporting appropriate referrals and maximising outcomes of patients who ultimately go on to receive SCS. It is hoped this will promote the efficient and optimal use of resources. Combined with the need to reduce treatment delays, the e-tool may also reduce unnecessary trial procedures and more effectively address the needs of patients requiring SCS.

### Study strengths and limitations

To our knowledge, this is the first survey exploring how the COVID-19 pandemic has impacted chronic pain patients on a neuromodulation waiting list in the United Kingdom. Importantly, these findings are specific to SCS, providing a focussed account of how this patient cohort is coping with their symptoms during the pandemic. However, as these findings are based on data collected during the first 2 months of the UK lockdown in a specific group of patients, it is crucial to repeat surveys of this kind in this and other patient groups. Of course, these data need to be substantiated internationally, which will hopefully aid with overcoming challenges associated with providing quality healthcare during public health crises. From a methodological perspective, the telephone nature of the survey presented advantages over more traditional paper-based approaches. This avoided potential reluctance on behalf of the patient to handle paper and reduced response time, potentially contributing to the high response rate (82%). Despite positive views towards attending for surgery, patients were not advised about the precise and as yet unquantified serious risks of elective surgery prior to responding to the survey. Therefore, the high rate of participants stating a willingness to attend for their procedure may not be an accurate reflection once patients and clinicians are more reliably informed with more published data. This is compounded by the fact that since the COVID-19 pandemic is a rapidly changing situation, it is difficult for healthcare professionals to find clarity about the risks of elective surgery and indeed care for patients with pain during these times.^40^ Of course, with research investigating the ongoing situation, it is hoped that greater clarity can be achieved in order to maximise patient safety while fulfilling their healthcare needs.

## Conclusion

Findings from this prospective telephone survey revealed that most chronic pain patients on an SCS waiting list experienced deteriorations in pain and mental health during the COVID-19 pandemic. Although some patients reported no adverse effects on ability to self-manage pain, increases in pain medication and reliance on support networks were acknowledged. The prospect of SCS surgery was well-received, with most agreeing to undergo testing, self-isolation, attending on the day of the procedure and preferring the surgery to happen as soon as possible. These findings suggest that even during unprecedented times, the clinical need for SCS in chronic pain patients is strong.

## Supplementary Material

Supplementary material
