# Supplementary material for: The impact of the COVID-19 pandemic on patients awaiting spinal cord stimulation surgery in the United Kingdom: a multi-centre patient survey
Source: Br J Pain. 2020 Aug 21;15(3):282–90. doi: 10.1177/2049463720948092 (PMC7443576; doi:10.1177/2049463720948092)
Supplement: Supplementary material [file SupplementalMaterial.pdf]

SUPPLEMENTARY SECTION

SCS waiting list survey

1. Age:
2. Sex:
3. Diagnosis awaiting SCS:
4. COVID-19 risk:
    a. Very High Risk. Defined as:
        i. Have had an organ transplant.
        ii. Are having chemotherapy or antibody treatment for cancer, including immunotherapy.
        iii. Are having an intense course of radiotherapy (radical radiotherapy) for lung cancer.
        iv. Are having targeted cancer treatments that can affect the immune system (such as protein kinase inhibitors or PARP inhibitors).
        v. Have blood or bone marrow cancer (such as leukaemia, lymphoma or myeloma).
        vi. Have had a bone marrow or stem cell transplant in the past 6 months, or are still taking immunosuppressant medicine.
        vii. Have been told by a doctor that you have a severe lung condition (such as cystic fibrosis, severe asthma or severe COPD).
        viii. Have a condition that means you have a very high risk of getting infections (such as SCID or sickle cell).
        ix. Are taking medicine that makes you much more likely to get infections (such as high doses of steroids).
        x. Have a serious heart condition and you're pregnant.
        xi. If you're at very high risk from coronavirus, you should have received a letter from the NHS.
    b. High Risk. Defined as:
        i. Are 70 or older.
        ii. Are pregnant.
        iii. Have a learning disability, a lung condition that's not severe (such as asthma, COPD, emphysema or bronchitis), heart disease (such as heart failure), high blood pressure (hypertension), diabetes, chronic kidney disease and/or liver disease (such as hepatitis).
        iv. Have a condition affecting your brain or nerves (such as Parkinson's disease, motor neurone disease, multiple sclerosis, or cerebral palsy).
        v. Have a problem with your spleen or have had your spleen removed.
        vi. Have a condition that means you have a high risk of getting infections (such as HIV, lupus or scleroderma).
        vii. Are taking medicine that can affect your immune system (such as low doses of steroids),
        viii. Are very obese (a BMI of 40 or above).
    c. Low Risk
5. How do you rate your pain in the last week?
    a. No pain
    b. Mild pain
    c. Moderate pain
    d. Severe pain
6. Has the severity of your pain changed during the COVID-19 crisis?
    a. Yes
    b. No

7. To what extent has your pain changed?
    a. Very much improved
    b. Much improved
    c. Minimally improved
    d. No change
    e. Minimally worse
    f. Much worse
    g. Very much worse
8. Has your mental health been impacted due to your pain symptoms during the COVID-19 crisis?
    a. Yes
    b. No
9. To what extent has your mental health changed?
    a. Very much improved
    b. Much improved
    c. Minimally improved
    d. No change
    e. Minimally worse
    f. Much worse
    g. Very much worse
10. Has your consumption of pain medication changed during the COVID-19 lockdown period?
    a. Reduced
    b. Stayed the same
    c. Increased
11. Has your reliance on a formal carer network or family increased during the lockdown due to increased pain severity?
    a. Yes
    b. No
12. Has the lockdown adversely affected your ability to manage your pain symptoms?
    a. Yes
    b. No
13. If we can schedule your procedure within the next 4 weeks (In the current COVID-19 situation, this is impossible to predict as it can take months) would you be happy to:
    a. Attend for COVID swabs 2-3 days before your procedure?
        i. Yes
        ii. No
    b. Self-isolate after the swab until procedure and after the procedure?
        i. Yes
        ii. No
    c. Attend for surgery on the day of your procedure?
        i. Yes
        ii. No

If the answer is NO, reason:

14. The procedure will be done predominantly under local anaesthetic. You might be given some strong pain killers, but not heavy sedation. Are you willing to undergo the procedure?
    a. Yes
    b. No
15. If possible and safe (your procedure will be done in the Non-COVID part of the hospital), would you prefer your procedure and sent home the same day?
    a. Yes

    b. No

If the answer is NO, Reason:

16. If you are willing to wait for surgery, how long would you prefer to wait for your surgery?
17. Have you had any signs and symptoms of COVID-19?
    a. Yes
    b. No
18. Have you had a COVID-19 test?
    a. Not tested
    b. Tested Positive
    c. Tested Negative
